# Effects of calorie intake and sampling time on thyroid stimulating hormone concentration

**DOI:** 10.1186/s12902-022-01005-7

**Published:** 2022-04-01

**Authors:** Aimei Dong, Youyuan Huang, Yucheng Huang, Bing Jia

**Affiliations:** 1 Department of Endocrinology, Peking University First Hospital, Beijing, 100034 China; 2 Department of General Practice, Peking University First Hospital, Beijing, 100034 China

**Keywords:** TSH, Sampling time, Fasting, Postprandial state, Thyroid function measurement

## Abstract

**Background:**

This study aimed to investigate the effects of blood sampling after calorie intake on thyroid stimulating hormone (TSH) level, compared with blood sampling in fasting state.

**Methods:**

This was a prospective, randomized, controlled study. Subjects from the outpatients in the department of endocrinology without evidence of thyroid diseases were included and then randomized into the fasting group, diet intake group, and glucose intake group, respectively. Fasting blood was collected from all subjects at 7:00 am for the measurement of TSH and free thyroxine (FT_4_) concentrations. Afterwards, the subjects were maintained at fasting state (fasting group), had an intake of the mixed diet with 400 kcal calories (diet intake group), and had an intake of 75 g glucose (glucose intake group), respectively, and blood was collected again 2 h later (9:00 am on the same day) for TSH and FT_4_ level measurement and comparison.

**Results:**

A total of 150 subjects were enrolled, of whom 146 met the inclusion criteria, with 48, 48, and 50 in the diet intake group, glucose intake group, and fasting group, respectively. The TSH in the diet intake group was significantly lower at 9:00 am (TSH_9am_) than the level at 7:00 am (TSH_7am_) (*P* < *0.001*), with a median variation of -0.71 mU/L, and a median variation rate of -32.4%. In the glucose intake group, TSH_9am_ was also significantly lower than TSH_7am_ (*P* < *0.001*), with a median variation of -0.73 mU/L, and a median variation rate of -31.5%. For the fasting group, TSH_9am_ decreased slightly but was significantly lower than TSH_7am_ (*P* < *0.001*), with a median variation of -0.1 mU/L, and a median variation rate of -5.2%. According to TSH_7am_ measurements, 9 subjects (3 subjects in each group) met the diagnostic criteria of subclinical hypothyroidism. However, according toTSH_9am_ measurements, only 2 patients in the fasting group met the diagnostic criteria of subclinical hypothyroidism.

**Conclusion:**

Compared with the fasting state, the TSH level at 2 h after the calorie intake was decreased by about 30%, which might influence the diagnosis of subclinical hypothyroidism.

**Trial registration:**

ChiCTR2100047454 (18/06/2021).

## Background

Thyroid-stimulating hormone (TSH) synthesized by the anterior pituitary gland, regulates the synthesis and secretion of thyroid hormones, and is also subject to feedback regulation of thyroid hormone concentration [1]. The guidelines of American and European thyroid associations [2, 3] have recommended using TSH level as the indicator to screen thyroid dysfunctions. The earliest changes in patients with primary thyroid diseases include alteration in TSH level. For instance, patients with subclinical thyroid dysfunctions are only show TSH level changes, characterized by TSH elevation in subclinical hypothyroidism and TSH reduction in subclinical hyperthyroidism. Subclinical hypothyroidism is associated with dyslipidemia, hypertension, metabolic syndrome [4], adverse maternal–fetal outcomes [2], and possible cardiovascular diseases [5]. TSH is also the major indicator for deciding whether treatments should be initiated in patients with subclinical hypothyroidism, as well as for evaluating whether the intervention goal has been achieved. Therefore, accurately measuring TSH levels has critical clinical significance.

Previous studies have reported that TSH secretion and its blood level display a circadian rhythm [6–11]. The relative amplitude (maximum absolute value of a periodically varying quantity) of TSH in one day can be as high as 36%. The variation of TSH during daytime is relatively small, but the food intake could influence the TSH level [12–17]. However, there are no special guideline recommendations on the time of blood sampling for TSH concentration measurement, as well as fasting requirement [2, 3]. In addition, the sampling time and the fasting state in evaluating the reference range of thyroid hormone by laboratories are generally ignored in clinical practice. Analysis of clinical laboratory data has shown that blood samples were delivered from 7:00 am to 4:00 pm for TSH level measurement, and the distribution patterns of TSH concentrations measured at different times differ significantly [18]. Our previous pilot studies (data not published) showed that the TSH level measured 2 h after breakfast (9:00 am), comparing with the fasting blood samples collected in the morning (7:00 am) was significantly reduced by about 30%, and there was a significant difference in the diagnosis of subclinical hypothyroidism between the two sampling methods.

Due to the circadian rhythm of blood TSH levels, it is necessary to further investigate the difference blood TSH concentration between 7:00 am and 9:00 am after meal to determine whether it is influenced by the food intake or diurnal rhythm. In addition, we aimed to evaluate the influence of different food components on blood TSH levels. Therefore, this study explored the changing trend of blood TSH level in subjects after maintaining fasting state or after the intake of calories provided by different nutrients, compared with the morning fasting state.

## Methods

### Subjects

The subjects were selected from the outpatients in the Department of Endocrinology at Peking University First Hospital between February 2021 and March 2021. The inclusion criteria were as follows: 1) patients aged 20–80 years; 2) subjects without an evident history of thyroid diseases; and 3) patients whose physical examinations showed no sign of thyroid enlargement. The exclusion criteria were as follows: 1) patients with acute infectious diseases; 2) subjects showing abnormal liver functions with elevated levels of ALT and/or AST to rising above 2.5 folds the upper limit of the reference range; 3) patients with chronic renal diseases with the serum creatinine level of > 130 μmol/L; 4) subjects treated with glucocorticoids, contraceptive pills, or other hormones; 5) subjects with the history of hypothalamic-pituitary diseases; 6) pregnant women. The study was approved by the Ethics Committee of Peking University First Hospital, and all participants signed the informed consent. The registration number of the study was ChiCTR2100047454.

### Randomization of subjects

SPSS statistics were used to generate random numbers (random number seeds: 20,000,000), which was used for 1:1:1 randomization of the subjects into 3 groups, namely diet intake group, glucose intake group, and fasting group.

### Interventions

For the subjects who participated in this study, the first blood sample was collected after fasting for over 10 h (about 7:00 ~ 7:30 am). Afterwards, subjects in the diet intake group were asked to intake mixed diets (with the calories of about 400 kcal), the time was recorded from the first bite of food, and the blood was collected again 2 h later (about 9:00 ~ 9:30 am). Subjects in the glucose intake group were asked to intake 75 g glucose (300 kcal calories), the time was recorded from the first sip of glucose solution, and the blood was collected again 2 h later (about 9:00 ~ 9:30 am). Subjects in the fasting group maintained a fasting state, and the blood was collected again 2 h later (about 9:00 ~ 9:30 am).

### Endpoints

The variation of TSH levels after fasting for 2 h or at 2 h after calorie intake, compared with the baseline level at 7:00 am, as well as the influence of blood collection time on the diagnosis of thyroid dysfunctions were the endpoints of this study.

### Measurement protocols

The blood serum samples were stored at -20 °C, and the TSH and FT_4_ concentrations were measured in the same sample. The TSH and FT_4_ levels were measured by the acridinium ester chemiluminescence assay using the Siemens Centaur XP machine. The sensitivity of measuring TSH was 0.019 mU/L, the intra-batch variable coefficient was 1.98%, and the reference range was 0.55–4.78 mU/L. The sensitivity of measuring FT_4_ was 1.3 pmol/L, the intra-batch variable coefficient was 1.95%, and the reference range was 11.48–22.70 pmol/L.

### Statistical methods

Sample size estimation: The finding of the pilot study showed that the reduction rate was 30% in the diet intake group and glucose intake group and 7% in the fasting group. The online tool http://powerandsamplesize.com/Calculators/ was utilized to calculate the sample size. One-way-ANOVA-pairwise mode for the comparison of means among multiple groups was used for the sample size estimation, with the β = 0.1, α = 0.05, and the number of groups of 3. The findings showed that 46 subjects were required for each group. Therefore, 50 subjects were planned to be included in each group in this study.

SPSS 24.0 software (IBM, Armonk, NY, USA) was used for data processing and statistical analysis. Kolmogorov normality test was performed for continuous data; the continuous data in normal distribution were described with means ± standard deviations (SD), while continuous data that were not in normal distribution were described with medians (interquartile ranges). For the comparison of the levels between two time points of blood sampling within the same group, paired t-test was used for analyzing the data with normal distribution, and paired Wilcoxon signed-rank test was used for analyzing the data with abnormal distribution. For the comparisons among 3 groups, one-way analysis of variances (one-way ANOVA) followed by post-hoc Bonferroni test was used for analyzing the data with normal distribution, and Kruskal–Wallis one-way ANOVA was used for analyzing the data without normal distribution. Bonferroni test was used to correct the significance for pair-wise comparisons. Linear regression was performed using TSH variation amplitude as the dependent factor. *P* < 0.05 (two-sided) was considered statistically significant. GraphPad Prime 7.04 software was used for plotting the figures.

## Results

### General characteristics of the subjects

A total of 150 subjects participated in this study, of which 4 were excluded due to a high serum creatinine level of > 130 μmol/L. Finally, 146 subjects including 70 males and 76 females, met the inclusion criteria. Forty-eight, 48, and 50 of the subjects were randomly included in the diet intake group, glucose intake group, and fasting group, respectively. The sex, age, liver function, and renal function of the subjects in the 3 groups were not significantly different (Table 1).

**Table 1:** General characteristics and thyroid function of the subjects in 3 groups at 7:00 am

	Diet intake group	Glucose intake group	Fasting group	*P* -value (among 3 groups)
n	48	48	50	
Sex (M/F)	25/23	20/28	25/25	0.557
Age (Years)	60.0 (51.0, 65.0) (29,76)	59.0 (50.0, 64.3) (29,72)	59.0 (49.8, 63.5) (28,79)	0.914
ALT (IU/L)	18.5 (14.0, 31.3)	23.0 (16.0, 27.0)	19.0 (14.0, 26.8)	0.968
AST (IU/L)	19.0 (15.0, 32.3)	20.0 (16.0, 23.5)	21.0 (17.0, 23.8)	0.857
Serum creatinine (μmol/L)	74.8 (61.9, 81.6)	81 (71, 89.6)	80.4 (64.7, 88.8)	0.169
TSH_7am_ (mU/L)	2.3 (1.4, 2.9)	2.2 (1.5, 3.1)	1.9 (1.3, 3.0)	0.644
FT_4 7am_ (pmol/L)	16.8 ± 2.0	16.2 ± 2.0	15.8 ± 2.1**	0.01

Sex was compared by Pearson’s chi-squared test among groups

FT_4_ was described with mean ± SD and compared by one-way ANOVA

Age was described with median values (range), other continuous non-normally distributed variables were presented as medians (P25, P75) and compared by Kruskal–Wallis test

**P < 0.001, comparing with diet-intake group by post-hoc Bonferroni test

The baseline TSH level at 7:00 am was 2.3 (1.4, 2.9) mU/L, 2.2 (1.5, 3.1) mU/L, and 1.9 (1.3, 3.0) mU/L in the diet intake group, glucose intake group, and fasting group, respectively, and the differences among the 3 groups were not statistically significant (*P* = 0.628).

The baseline FT4 level at 7:00 am was 16.8 ± 2.0 pmol/L, 16.2 ± 2.0 pmol/L, and 15.8 ± 2.1 pmol/L in the diet intake group, glucose intake group, and fasting group, respectively. The differences among the three groups were statistically significant (*P* = 0.01). Post-hoc Bonferroni test was found statistically significant between the diet intake group and fasting group (*P* < 0.001).

### TSH level 2 h after fasting or calorie intake

In the diet intake group, the TSH level at 9:00 am (TSH_9am_) was significantly lower than that at 2 h before (TSH_7am_) the fasting state (TSH_7am_ vs TSH_9am_: 2.3 (1.4, 2.9) vs 1.5 (1.0, 2.1), *P* < 0.001). The median of TSH variation(TSH variation = TSH_9am_-TSH_7am_) was -0.71 mU/L (95% CI: -0.90, -0.61 mU/L), and the median of TSH variation rate (TSH variation rate = (TSH_9am_-TSH_7am_)/ TSH_7am_)) was -32.4% (95% CI: -34.6, -27.5%).

In the glucose intake group, the TSH level at 9:00 am (TSH_9am_) was significantly lower than that at 2 h before (TSH_7am_) the fasting state (TSH_7am_ vs TSH_9am_: 2.2 (1.5, 3.1) vs 1.4 (0.9, 2.1), *P* < 0.001). The median of TSH variation was -0.73 mU/L (95% CI: -1.01, -0.68 mU/L), and the median of TSH variation rate was -31.5% (95% CI: -35.8, -30.4%) (Table 2).

**Table 2:** Influence of fasting and calorie intake on TSH level

Median (P25, P75)	Diet intake group	Glucose intake group	Fasting group	*P* value
TSH_7am_ (mU/L)	2.3 (1.4, 2.9)	2.2 (1.5, 3.1)	1.9 (1.3, 3)	0.664
TSH_9am_ (mU/L)	1.5 (1, 2.1) ^***^	1.4 (0.9, 2.1) ^***^	1.7 (1.2, 3.1) ^***^	0.094
TSH variation (mU/L)	-0.71 (-1.02, -0.36) ^###^	-0.73 (-1.05, -0.44) ^&&&^	-0.1 (-0.26, 0.04)	< 0.001
TSH variation rate (%)	-32.4 (-39.3, -25.3) ^###^	-31.5 (-40.7, -24) ^&&&^	-5.2 (-16.1, 1.6)	< 0.001

Variables were presented as medians (P25, P75) and compared by Kruskal–Wallis test among groups

^***^ *P* < 0.001, comparing withTSH_7am;_^###^ *P* < 0.001, comparing with the fasting group; ^&&&^ P < 0.001, comparing with the fasting group

TSH variation = TSH_9am-_ TSH_7am;_ TSH variation rate = (TSH_9am-_ TSH_7am_)/ TSH_7am;_

In the fasting group, the TSH level at 9:00 am (TSH_9am_) was slightly but significantly lower than that at 2 h before (TSH_7am_) the fasting state (*P* < 0.001). The median of TSH variation was -0.1 mU/L (95% CI: -0.27, 0.05 mU/L), and the median of TSH variation rate was -5.2% (95% CI: -11.7, 1.9%) (Table 2).

The variation and variation rate of the TSH levels were significantly different among the three groups (*P* < 0.001). The pair-wise comparison showed that the TSH variation and variation rate were not significantly different between the diet intake group and glucose intake group. In contrast, the TSH variation and TSH variation rate in the fasting group were significantly lower than those in the diet intake group (*P* < 0.001 after Bonferroni correction) and the glucose intake group (*P* < 0.001 after Bonferroni correction) (Table 2).

### Association between TSH variation and fasting TSH_7am_ level

The TSH variation was significantly negatively correlated with the TSH_7am_ level (Spearman rank correlation coefficient: -0.551 for all subjects, -0.809 for diet intake group, -0.881 for glucose intake group, and -0.135 for fasting group).

**Fig. 1:**
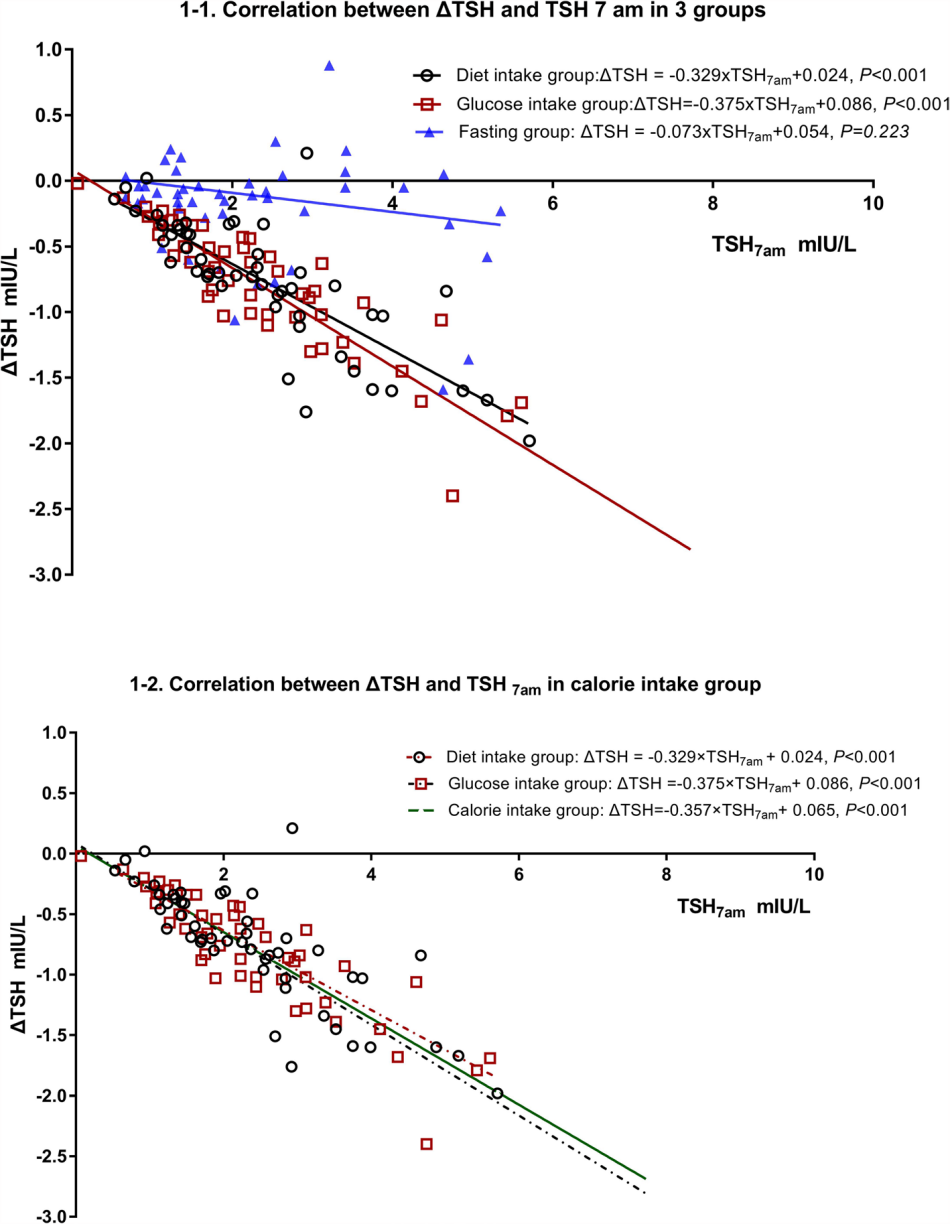
Correlation between TSH variation and fasting TSH_7am_ level in linear regression model. *Calorie intake group: data in the diet intake group and glucose intake group were combined together. TSH7am: TSH level at 7am, ΔTSH = TSH9am—TSH7am

Single-factor linear regression was performed using the TSH_7am_ as the independent factor, and the TSH variation as the dependent factor (Fig. 1–1). The regression coefficient and determination coefficient (R^2^) were -0.329 (95% CI: -0.402, -0.255, *P* < 0.001) and 0.637 in the diet intake group, -0.375 (95% CI: -0.417, -0.311, *P* < 0.001) and 0.835 in the glucose intake group, and -0.073 (95% CI: -0.124, 0.337, *P* = 0.223) and 0.026 in the fasting group, respectively.

The comparison between the diet intake group and glucose intake group showed that the TSH variation was not significantly different after calorie intake. The linear regression using the fasting TSH as the independent factor also showed that the regression coefficient and intercept were not significantly different between the two groups. Therefore, data of these two groups were combined as the calorie intake group, and then linear regression analysis was performed for TSH variation and TSH_7am_ (Fig. 1–2). The results showed that the regression coefficient was -0.357 (95% CI: -0.402, -0.255, *P* < 0.001), and R^2^ was 0.748. These findings suggested that 74% of the TSH variation was determined by TSH_7am_.

The partial correlation analysis with TSH_7am_ controlled showed that TSH variation amplitude was not significantly correlated with age and fasting FT_4_.

### Influence of blood sample collection at different times on the evaluation of thyroid functions

According to the TSH and FT_4_ levels measured at 7:00 am and 9:00 am, no subjects included in this study had clinical thyroid dysfunctions.

Subjects with TSH concentration of > 4.78 mU/L and FT_4_ level in the reference range were diagnosed with subclinical hypothyroidism. According to the levels measured at 7:00 am in the fasting state, 3 subjects in each group met the diagnostic criteria of subclinical hypothyroidism. However, according to the TSH level measured at 9:00 am after maintaining fasting or after calorie intake, the number of subjects diagnosed with subclinical hypothyroidism decreased. Specifically, no subjects in the diet intake group or glucose intake group were diagnosed with subclinical hypothyroidism, and only 2 subjects in the fasting group were diagnosed with subclinical hypothyroidism.

Subjects with TSH concentration of < 0.55 mU/L and FT_4_ level in the reference range were diagnosed with subclinical hyperthyroidism. According to the levels measured at 7:00 am in the fasting state, 1, 1, and 0 subjects in the diet intake group, glucose intake group, and fasting group met the diagnostic criteria of subclinical hyperthyroidism, respectively. However, according to the TSH level measured at 9:00 am after maintaining fasting or after calorie intake, the number of subjects diagnosed with subclinical hyperthyroidism increased. Specifically, 1, 2, and 1 subjects in the diet intake group, glucose intake group, and fasting group met the diagnostic criteria of subclinical hyperthyroidism, respectively (Table 3).

**Table 3:** Number of subjects with subclinical thyroid dysfunctions according to the measurements at different time points

N (%)		Diet intake group (*n* = 48)	Glucose intake group (*n* = 48)	Fasting group (*n* = 50)	Total
Subclinical hypothyroidism	7 am (fasting state)	3 (6.3%)	3 (6.3%)	3 (6.0%)	9 (6.2%)
	9 am (2 h after fasting or calorie intake)	0 (0%)	0 (0%)	2 (4.0%)	2 (1.4%)
Subclinical hyperthyroidism	7 am (fasting state)	1 (2.1%)	1 (2.1%)	0 (0%)	2 (1.4%)
	9 am (2 h after maintained fasting or calorie intake)	1 (2.1%)	2 (4.2%)	1 (2.0%)	4 (2.7%)

Numbers of euthyroid subjects were not shown

Distribution of thyroid function status was compared between 7 and 9 am by the chi-square test for ordinal categorical variable, *P* value: diet intake group 0.008, glucose intake group 0.028, fasting group 0.191

## Discussion

The findings of this study showed that the TSH level was reduced significantly by about 30% after calorie intake in the morning, The components of calories had no significant influence on TSH variation rate when the calories intake was similar. The TSH level was reduced slightly by 5.2% in the subjects after maintaining the fasting state. The rate of TSH reduction was significantly pronounced after calorie intake compare to the fasting state, suggesting that the influence of food on TSH was more evident than the diurnal rhythm of TSH.

Previous studies have reported that the TSH level has an apparent circadian rhythm [6–11], during which the level peaks at 2–4 am, reaching the lowest level at 3–8 pm. With the elapse of time in the morning, the TSH level tends to reduce. The early studies on the rhythm of TSH showed no influence of food intake on TSH level [10]. However, subsequent studies showed that food intake could influence the level of TSH measurements [12–17, 19], with a TSH reduction rate of 10–35%.

Many studies have only focused on the variation of TSH levels before and after food intake, but TSH is also influenced by circadian rhythm, and time-lapse can also influence the TSH level. However, only very few studies investigated whether food intake could influence TSH level independent of diurnal rhythm, and the findings were controversial. One study [14] including 20 subjects with normal thyroid functions showed that the serum TSH level at 60 min after lunch and supper intake (1061 kcal calorie) was significantly lower than that before the diet, and the reduction was more pronounced after lunch (median: -0.25 mU/L) compared to supper intake (-0.2 mU/L). The reduction amplitude of TSH was -0.1 mU/L after taking low-calorie food for lunch (212 kcal), which was lower than having high-calorie food (1061 kcal; reduction amplitude: -0.25 mU/L). These findings demonstrated that food intake could independently reduce the level of TSH, which was agreeable with our findings. However, the study did not investigate the influence of breakfast intake on the TSH level. In the present study, the TSH reduction was more pronounced after the breakfast intake (median value about -0.7 mU/L), which could be associated with the variation of TSH at different times due to diurnal rhythm. The TSH level tends to reduce in the morning, which is enhanced by the influence of the food intake; while the TSH level after dinner tends to increase, which can alleviate the influence of food intake. However, another study [17] showed that compared with the fasting state at 7–8 am, the amplitude of TSH reduction at 140 min after food intake of their own choices was similar to that after fasting for 140 min (-29.3% vs -28.3%). The differences in the findings could be associated with the variations in the time of blood sample collection, the time between the two blood samplings, and calorie intake from food.

In light of the findings of previous studies, we speculated that the TSH reduction after breakfast observed in this study could be combinedly influenced by food intake and sampling time (the diurnal rhythm of TSH). However, the influence of food intake seemed to be more prominent. The mechanism underlying the association between food intake and TSH are still unclear yet. We speculated that the reduction of TSH could be associated with the acute elevation of somatostatin level after food intake [20], as somatostatin could inhibit the synthesis and secretion of TSH [1, 11].

The findings of this study showed that the variation of TSH level after calorie intake in the morning might influence the diagnosis of subclinical thyroid dysfunction. Subjects with subclinical hypothyroidism might be underestimated due to the non-fasting state. In certain conditions, such as pregnancy, the ideal range of TSH is narrowed down, and the TSH value is required to decide the treatment strategy, while the variations of the TSH level could lead to significant clinical influences.

The limitations of this study could be as follows: all subjects in this study were outpatients and from the Department of Endocrinology of only one hospital, and the subjects had underlying diseases, such as diabetes, hypertension, and osteoporosis. The research covered a considerable age span with a median age of 60 years old. Though the variation of TSH measurements in the older population has limited significance in clinical practice, it is more significant to investigate the variation of TSH measurements among young people, especially for women with family planning. Unfortunately, such subjects were very rare in our study. The findings need to be further validated by future studies with higher representativeness, especially in subjects with a relatively narrow range of TSH level, such as pregnant women.The criteria were not clearly defined to exclude patients with chronic disease at risk of the euthyroid sick syndrome, which might affect the patients TSH levels. Another limitation of this study is the slight but significant variation in FT4 levels among the three groups at baseline. Furthermore, subjects in this study only consumed one level of calories, and the influences of food with different calories were not investigated.

## Conclusion

In summary, the TSH level was reduced significantly after food intake, compared with that at fasting state in the morning. If the reference range of TSH used in the laboratory was from fasting blood samples, it would be better to evaluate the TSH level in fasting blood obtained in the morning compared with random or postprandial samples.

## Data Availability

All data generated or analysed during this study are included in this published article.

## Abbreviation

TSH: Thyroid-stimulating hormone
